# Typical patterns of disordered eating among Swedish adolescents: associations with emotion dysregulation, depression, and self-esteem

**DOI:** 10.1186/s40337-016-0122-2

**Published:** 2016-11-04

**Authors:** Erika Hansson, Daiva Daukantaitė, Per Johnsson

**Affiliations:** 1 Department of Psychology, Lund University, Lund, Sweden; 2 Centre for Psychology, Kristianstad University, Kristianstad, Sweden

## Abstract

**Background:**

Using the person-oriented approach, we determined the relationships between four indicators (restraint and eating, shape, and weight concerns) of disordered eating (DE), as measured by the self-reported Eating Disorders Examination Questionnaire (EDE-Q), to identify typical DE patterns. We then related these patterns to clinical EDE-Q cut-off scores and emotion dysregulation, depression, self-esteem, and two categories of DE behaviors (≥2 or ≤1 “yes” responses on the SCOFF questionnaire).

**Method:**

Typical patterns of DE were identified in a community sample of 1,265 Swedish adolescents (*M*_*age*_ = 16.19, *SD* = 1.21; age range 13.5–19 years) using a cluster analysis. Separate analyses were performed for girls (*n* = 689) and boys (*n* = 576).

**Results:**

The cluster analysis yielded a six-cluster solution for each gender. Four of the six clusters for girls and five for boys showed scores above the clinical cut-off on at least one of the four DE indicators. For girls, the two clusters that scored above the clinical cut-offs on all four DE indicators reported severe psychological problems, including high scores on emotion dysregulation and depression and low scores on self-esteem. In contrast, for boys, although two clusters reported above the clinical cut-off on all four indicators, only the cluster with exceedingly high scores on shape and weight concerns reported high emotion dysregulation and depression, and extremely low self-esteem. Furthermore, significantly more girls and boys in the most problematic DE clusters reported ≥2 “yes” responses on the SCOFF questionnaire (as opposed to ≤1 response), indicating clear signs of DE and severe psychological difficulties.

**Conclusion:**

We suspect that the various problematic DE patterns will require different paths back to a healthy diet. However, more research is needed to determine the developmental trajectories of these DE patterns and ensure more precise clinical cut-off scores, especially for boys. Comprehensive understanding of DE patterns might be of use to healthcare professionals for detecting DE before it develops into an eating disorder.

**Trial registration:**

Lund, EPN (dnr: 2012/499).

## Plain English summary

We sought to identify groups of adolescents with distinct disordered eating patterns by looking at similarities in their restraint and eating, shape, and weight concerns. Then, we compared these subgroups in their levels of emotion dysregulation, depression, and self-esteem to clarify which subgroups had notable psychological problems. Identification of the different problematic disordered eating patterns for girls and boys can help healthcare personnel, such as school nurses, detect disordered eating among adolescents early on.

## Background

Disordered eating (DE) is a complex and multifaceted phenomenon [31] that can take many forms [7]. Waaddegaard et al. [46] defined DE rather broadly as the behaviors and attitudes with regard to body perception, eating habits, weight regulation, and self-evaluation and we have used this definition in the present study. DE is a predictive factor of eating disorders [35, 46], which can be fatal if left unchecked [42]. Therefore, it is of great importance to identify DE as early as possible.

Most research on DE has been dimensional-that is, it focused on the severity of the DE-as opposed to categorical (i.e., focused on distinct types of DE behavior). The debate over whether DE should be viewed as dimensional or categorical has raged for years, but both views seem desirable for representing the full spectrum of DE and detecting DE as early as possible [50]. Usually, DE has been studied by first examining a number of DE indicators, such as eating and/or shape concerns, and then relating either individual DE indicators or the total scores of those indicators to different psychosocial problems such as depression or self-esteem (e.g., [2, 40, 43]). A variable-oriented approach, built on the examination of linear relationships and mean differences, provides important knowledge about the relationships between various DE indicators and psychosocial difficulties. However, the knowledge provided can be rather segmented. In contrast, a person-oriented approach, which focuses on obtaining and studying information about the chosen sub-system (in this case, DE) as a whole, could be more informative. According to this approach, the key indicators of particular systems (e.g., DE) are regarded as indivisible and therefore must be studied and interpreted simultaneously [29, 30]. In the article, we draw on a person-oriented approach to identify subgroups of individuals with specific DE patterns because essential relationships in these data might not be reflected through ordinary correlational analyses due to their non-linearity. This can, to some degree, be compared to how clinical psychologists examine various different symptoms to build a specific profile for each patient to diagnose eating or other disorders.

Simultaneous examination of the different aspects of DE-including restraint and eating, shape, and weight concerns, as measured by the self-reported Eating Disorder Examination Questionnaire (EDE-Q; [12]), revised for adolescents [6]-is expected to yield a variety of different DE patterns. By examining those specific patterns, and comparing them in terms of their associations with emotion dysregulation, depression, and self-esteem, as well as two categories of DE behaviors (≥2 or ≤ 1 “yes” responses as measured by the SCOFF questionnaire), we expect to obtain a broader picture of the psychological problems found in specific problematic DE subgroups.

Furthermore, we also compare the DE indicators within each subgroup to clinical cut-off values of the four EDE-Q subscales suggested by Ekeroth and Birgegard [11] to better understand whether only those adolescents who score above the suggested clinical cut-off for all indicators are at risk of developing psychological problems or whether scoring above the cut-offs on only specific indicators presents this risk. Traditionally, a mean global score of 4.0 on the Eating Disorder Examination Interview has been used as a threshold for eating disorder psychopathology in community studies, but clinical evidence has shown that nearly half of the patients diagnosed with an eating disorder obtain a global score of less than 4.0 (e.g., [49]). It therefore has been suggested that a cut-off of 4.0 has limited clinical utility, and if utilized for screening purposes, might yield underestimates of the prevalence of ED [37]. Thus, using more sensitive cut-off scores of the four DE indicators, such as those suggested by Ekeroth and Birgegard [11], in addition to studying other psychological difficulties, could be important for the early prevention of DE because it would allow for identifying the early warning signs of DE. Ultimately, this may be useful for preventing eating problems that otherwise might become chronic into adulthood [39].

Gender is generally considered an important factor for understanding the etiology and maintenance of psychopathologies such as DE [24, 33, 36, 44]. In particular, DE is much more prevalent among females, although the reasons for this are unknown [19]. Interestingly, while the core psychopathology of DE has been suggested to be similar for men and women and related to an distorted evaluation of one’s shape and weight [13], its expression has appears to show gender differences [17, 33, 36]. Despite the appearance, these standpoints are not contradictory; nevertheless, more research is needed to understand them, especially among men and boys, who are underrepresented in most studies. In the present study, we were interested in examining whether boys have similar DE patterns to girls.

Emotion dysregulation, defined as an individual’s inability to optimize his or her emotional dynamics in response to the demands of the environment [1], is regarded as a significant contributor to the development of DE among girls aged 14–18 years [32]. In a study of 89 adults (approximately 90 % of which were women) with bulimia nervosa, a lack of acceptance of emotions, impulse control, and emotion regulation strategies were significantly correlated with the severity of the eating disorder [27]. Girls with high levels of DE also reported experiencing greater negative affect, difficulties with emotional awareness, and difficulty coping constructively with negative emotions than did girls with low levels of DE [41]. DE has also been linked with emotion dysregulation in young adult Australian men [16].

DE, regardless of the severity, is also correlated with depressive thoughts [9], while depressive symptoms at age 14 appear to increase eating disorder symptoms in Australian girls at age 20 [2]. Furthermore, depressive symptoms, other psychiatric disorders, and suicidality were all found to be associated with DE among American adolescents of both genders [43, 45], while negative affect is a good predictor of DE in young men [25].

Besides emotion dysregulation and depression, low self-esteem is a significant contributor to DE ([40] p. 1527): Adolescents (*M*_age_ = 16.31, *SD* = 1.07; 21.3 % male) with low self-esteem were found to be at greater risk of eating disorder symptoms as well as depression [8]. Relatedly, exaggerated views of shape and weight are partly mediated by low self-esteem [10], while a study of 320 Norwegian students (35 % men) showed that self-esteem had direct effects on restrained eating and compensatory behavior [5].

To summarize, while there is a plethora of studies on the various DE concerns and behaviors and their relations to psychological difficulties, our understanding of these relationships, and DE concerns in general, remains rather segmented. In this study, we examined the four main DE indicators-restraint and eating, shape, and weight concerns-together to identify typical patterns of DE in a community sample of Swedish adolescents. We then compared these patterns to the clinical cut-offs suggested by Ekeroth and Birgegard [11] and determined their relations with emotion dysregulation, depression, self-esteem, and two categories of DE behaviors (≥2 or ≤ 1 “yes” responses on the SCOFF) to identify which DE patterns are particularly problematic. We hypothesized that adolescents with non-problematic DE patterns (i.e., below the clinical cut-offs) would report more effective emotion regulatory capabilities, fewer depressive symptoms, and higher self-esteem, whereas adolescents with problematic DE patterns would report higher levels of emotion dysregulation, more severe depressive symptoms, and lower self-esteem. Identifying these problematic patterns is expected to benefit healthcare professionals, as it would broaden the evaluation of adolescent DE and thereby contribute to efforts to prevent DE from worsening at an earlier stage.

## Method

### Participants

This study was conducted in a municipality in southern Sweden between January and March 2014. The sample comprised 1,265 students (*M*_age_ = 16.19, *SD* = 1.21; age range 13.5–19 years, 54.5 % female), or approximately 78 % of the 1,621 students attending the schools participating in this study. Of the 356 students who did not participate, 62 refrained from participation either of their own or of their parents’ volition. The remaining 294 students were for various reasons absent from school on the day of the data collection.

In total, 83.1 % of the adolescents were born in Sweden or another Scandinavian country; the others were born in another European country (4.7 %), the Middle East (7.9 %), or other parts of the world (4.3 %). Approximately two-thirds of the parents were born in Sweden or another Scandinavian country (67.0 % of mothers and 67.7 % of fathers), with the rest being born in another European country (12.9 and 12.4 %, respectively), the Middle East (11.7 and 12.0 %), or other parts of the world (8.4 and 8.0 %).

Approximately three-quarters of the adolescents (74.8 %) lived in two-parent households with biological parents, while 10.3 % lived in a single-parent household (8.2 % with the mother) and 3.7 % lived with a parent and a stepparent (3.1 % with the biological mother). Roughly one in ten adolescents (9.9 %) lived alternatingly with mother and father, 0.9 % lived with adults other than their parents, and 0.5 % lived alone.

### Procedure

The legal guardians of students below age 15 and the students themselves, irrespective of age, received written information about the study aims and procedures, as well as their right to decline participation. The students were again informed on the day of the data collection and assured of their confidentiality. Parents provided their *passive consent*, which meant that they had to sign and return a form if they did not wish for their child to participate in the study. Students consented actively by completing the questionnaire, which took approximately one hour. The study was approved by the Regional Ethics Committee in Lund, Sweden.

### Measures

#### Disordered eating behaviors

##### EDE-Q

An updated version of the original 36-item EDE-Q [12] was used in the present study; this version comprises only 22 items and restricts the time range to 14 days to better suit adolescent populations [6]. The items that we used were from the following subscales: eating concern (e.g., “How many of the past 14 days have you had a definite fear of losing control over eating?”); restraint (e.g., “How many of the past 14 days have you been deliberately trying to limit the amount of food you eat to influence your shape or weight?”); shape concern (e.g., “How many of the past 14 days have you had a definite desire to have a totally flat stomach?”); and weight concern (e.g., “How many of the past 14 days have you had a strong desire to lose weight?”). Each item is answered on a 7-point Likert scale ranging from 0 (“no days”) to 6 (“every day”). The item scores in each subscale can be averaged to provide subscale scores, and then a global score can be calculated by averaging the subscale scores. Higher scores are indicative of more severe eating disorder psychopathology.

Ekeroth and Birgegard [11] have suggested various clinical cut-off points for the EDE-Q’s global and subscales scores. They used Jacobson and Truax’s [21] method of determining clinically meaningful changes by evaluating the clinical significance (i.e., an individual’s transition between a clinical/dysfunctional population and a normal/functional population based on an empirically derived cut-off point) and the reliable change index (i.e., the reliability of an instrument’s change score). The clinical cut-off points for the EDE-Q global and subscale scores for girls were as follows (boys’ cut-off scores are presented in parentheses): global, 2.17 (1.06), eating concern, 1.50 (.69), restraint, 1.88 (.92), shape concern 2.90 (1.45), and weight concern, 2.39 (1.25). The Cronbach’s alpha was .95 for the whole EDE-Q, .77 for eating concern, .90 for restraint, .93 for shape concern, and .85 for weight concern in the present study.

##### SCOFF

The SCOFF questionnaire [34] contains five items concerning eating habits and attitudes toward weight and body shape: “Do you make yourself sick (vomit) because you feel uncomfortably full?” “Do you worry that you have lost control over how much you eat?” “Have you recently lost more than one stone (15 pounds) [around 6.8 kg] in a 3-month period?” “Do you believe yourself to be fat when others say you are thin?” and “Would you say that food dominates your life?” A threshold of two positive answers is often used to indicate a suspected eating disorder [28, 34]. The SCOFF has been validated among Swedish adolescents [17].

#### Emotion dysregulation

##### Emotion dysregulation

The Difficulties in Emotion Regulation Scale (DERS; [15]) is a 36-item self-report questionnaire that has been validated for adolescents [48]. The DERS comprises six dimensions of emotion regulation; (1) lack of emotional *awareness* (e.g., “I am attentive to my feelings” [reverse scored]); (2) lack of emotional *clarity* (e.g., “I have no idea how I am feeling”); (3) *impulse control* difficulties (e.g., “When I’m upset, I feel out of control”); (4) difficulties in engaging in *goal* directed behaviors (e.g., “When I’m upset, I have difficulties getting work done”); (5) *non-acceptance* of emotional responses (e.g., “When I’m upset, I feel guilty for feeling that way”); and (6) limited access to emotion regulation *strategies* (e.g., “When I’m upset, my emotions feel overwhelming”). The items are rated on a five-point Likert scale ranging from (1) “almost never” to (5) “almost always” and the participants are asked to rate how frequently each statement applies to them. The DERS has demonstrated high internal consistency in the past (α = .93; [15]). In the current study, the Cronbach’s alphas were .94 for the DERS total scale, .76 for awareness, .81 for clarity, .85 for impulsivity, .86 for goals, .87 for non-acceptance, and .86 for strategies.

#### Psychological health

##### Depression

The Center for Epidemiological Studies Depression Scale for Children (CESD-C) [14] is a 20-item measure for assessing depressive symptoms in adolescents. The scale covers six broad symptom areas including (1) sleep disturbances, (2) guilt/worthlessness. (3) helplessness/hopelessness, (4) psychomotor retardation, (5) loss of appetite, and (6) positive mood (which is reverse scored). For each item, the respondent indicates the extent to which he or she has felt this way in the past week using a four-point Likert scale that ranges from 0 (“not at all”) to 3 (“all the time”). Higher scores indicate more severe depressive symptoms. In the current study, the Cronbach’s alpha was .91.

##### Self-esteem

Lack of self-esteem was measured using the 10-item Rosenberg Self-Esteem Scale [38] that measures global self-worth by measuring both positive and negative feelings about the self. All items are answered using a four-point Likert scale format ranging from strongly agree to strongly disagree. Example items for this scale include “I feel that I am a person of worth, at least on an equal plane with others.” The Cronbach’s alpha in this study was .90.

#### Statistical analyses

A cluster analysis within the framework of the LICUR procedure [3] was used to identify the various DE profiles. We used the SLEIPNER statistical package [4] to perform the cluster analysis. The cluster analysis performed with SLEIPNER has several advantages over traditional forms of cluster analysis, including the ability to analyze the explained variance of cluster solutions and homogeneity coefficients of the clusters, and the fact that it contains an explicit procedure for testing the statistical significance of the cluster solution (using Monte Carlo simulations to create random data for comparison).

The cluster analysis was carried out in three steps. First, multivariate outliers were identified and removed via the RESIDUE module. Second, the remaining subjects were cluster analyzed using the agglomerative hierarchical method [47]. Four criteria were used to establish an appropriate number of clusters to extract: (a) the cluster solution must have theoretical meaning; (b) a pronounced drop in the explained error sum of squares (EESS; explained below) should occur when a cluster solution with one less cluster is extracted; (c) the number of clusters should not be more than 15 and should not be less than five; and (d) the size of the EESS for the chosen cluster solution should preferably be no less than 67 %, and at the very least should exceed 50 % [3]. Finally, a data simulation was carried out to verify that the EESS was higher than could be expected by chance using a random data set.

An evaluation of the trustworthiness and explanatory power of the clusters was based on their degree of homogeneity. The average squared Euclidean distances (ASEDs) were computed between all members within a cluster (the homogeneity coefficient, *hc*). For the total cohort, or a one-cluster solution, the *hc* is 2.00 for standardized variables. As a rule of thumb, a value below 1.00 for a cluster is considered highly desirable and a value below 0.50 indicates a reasonably homogenous cluster. Finally, we assessed the differences in the various validation variables (i.e., SCOFF, emotion dysregulation, depression, and self-esteem) between the clusters. If the classification structure is valid and useful, then clear differences in the expected directions should be found between the clusters in these three variables.

## Results

### Correlational analyses

To evaluate the relationships between the DE indicators-restraint and eating, shape, and weight concerns-used for the cluster analyses, we conducted bivariate correlation analyses separately for boys and girls. As Table 1 shows, highly significant positive correlations were obtained between all indicators for both genders. Particularly high inter-correlations were obtained between the weight and shape concern subscales scores for both girls (*r* = .91) and boys (*r* = .90).

**Table 1:** Mean (SD) and Bivariate inter-correlations between the EDE-Q subscales for Girls and Boys

The EDE-Q subscales	M (SD)		Eating Concern		Restraint		Shape Concern	
	girls	boys	girls	boys	girls	boys	girls	boys
Eating Concern	0.69 (1.01)	0.23 (0.57)	-	-	-	-	-	-
Restraint	0.91 (1.24)	0.43 (0.94)	.67	.52	-	-	-	-
Shape Concern	1.83 (1.62)	0.63 (0.97)	.78	.73	.67	.60	-	-
Weight Concern	1.48 (1.47)	0.60 (0.95)	.77	.70	.66	.60	.91	.90

*Note:* All reported correlations are significant at *p* < .001. All mean differences were significant at *p* < .001. N varies between 414–650 for girls and 325–521 for boys

### Cluster analyses

Following the rationale outlined above, we performed two cluster analyses (on the samples of 666 girls and 538 boys) using the four DE indicators. Both cluster analyses yielded a six-cluster solution with explained variances of 80.05 % for girls and 76.16 % for boys. Table 2 shows the mean profiles for the cluster solutions and their suggested labels.

**Table 2:** M (SD) of the EDE-Q Subscales for Girls’ and Boys’ Clusters

			EDE-Q Subscales								EDE-Q Global	
	*n*		Eating		Restraint		Shape		Weight			
Clusters	Girls	Boys	Girls	Boys	Girls	Boys	Girls	Boys	Girls	Boys	Girls	Boys
1. Non-problematic	331	407	.12 (.23)	.06 (.18)	.19 (.33)	.07 (.20)	.58 (.50)	.19 (.23)	.33 (.33)	.20 (.30)	.30 (.23)	.13 (.15)
2. Elevated shape & weight (girls) Clinical shape & elevated weight (boys)	143	56	.47 (.44)	.15 (.18)	.55 (.51)	.29 (.37)	2.06 (.83)	**1.45** (.56)	1.78 (.80)	1.24 (.77)	1.20 (.39)	.77 (.29)
3. Clinical restraint	37	20	.39 (.27)	.10 (.14)	**2.23** (.58)	**1.77** (.51)	2.15 (.88)	.56 (.40)	1.78 (.78)	.62 (.42)	1.61 (.37)	.76 (.23)
4. Clinical shape and weight (girls) Clinical DE* (boys)	55	27	1.23 (54)	**1.23** (.56)	1.26 (.65)	**1.10** (.77)	**3.90** (.84)	**2.03** (.63)	**3.08** (.91)	**1.90** (.69)	**2.36** (.38)	**1.53** (.34)
5. Clinical DE*, high shape & weight	61	8	**2.87** (.77)	**2.69** (.38)	**2.23** (.84)	**1.69** (1.26)	**4.54** (.86)	**4.03** (.89)	**4.03** (.94)	**4.15** (.60)	**3.44** (.52)	**2.97** (.59)
6. Clinical DE*, high restraint	39	20	**2.19** (.97)	.44 (.51)	**4.27** (1.01)	**2.82** (.85)	**3.98** (1.17)	**1.98** (.69)	**3.46** (.99)	**1.62** (.85)	**3.54** (.78)	**1.76** (.51)
Total	666	538										

Note: *Clinical DE used as a description when *M* values on all subscales as well as on the EDE-Q Global scale are above clinical cut-off norm values suggested by Ekeroth & Birgegard [11]. *Values above clinical cut-off points in bold*

#### Cluster solutions for girls

Cluster G1 (*n* = 331; 50 %) was interpreted as the *non-problematic* cluster, which included girls who reported low scores on all EDE-Q subscales (and thus indicating low levels of DE). Cluster G2 (*n* = 143; 21 %) was characterized by elevated scores for shape and weight concerns, and thus was labeled the *elevated shape and weight concern* cluster. Cluster G3 (*n* = 37; 6 %) also had elevated shape and weight concern scores, along with restraint scores that exceeded the clinical cut-off point suggested by Ekeroth and Birgegard [11]; as such, this cluster was labeled the *clinical restraint* cluster. Cluster G4 (*n* = 55; 8 %) was interpreted as the *clinical shape and weight concern* cluster because it exhibited shape and weight concern scores above the clinical cut-off point. Cluster G5 (*n* = 61; 9 %) was termed the *clinical DE with high shape and weight concern* cluster because all of the EDE-Q subscales were above the clinical cut-off points, with the shape and weight concern scores being particularly high. Finally, Cluster G6 (*n* = 39; 6 %) also showed scores above the clinical cut-off on all subscales, but instead showed especially high scores for restraint; therefore, it was defined as the *clinical DE with high restraint* cluster. All of the clusters for girls except for the *clinical DE with high restraint* cluster (*hc* = 1.32) indicated high cluster homogeneity.

#### Cluster solutions for boys

Cluster B1 (*n* = 407; 76 %) was defined as the *non-problematic* cluster because it showed low scores on all subscales. Cluster B2 (*n* = 56; 10 %) was characterized by shape concern scores above the clinical cut-off point as well as heightened weight concern scores; it was thus termed the *clinical shape and elevated weight concern* cluster. Cluster B3 (*n* = 20; 4 %) displayed restraint subscale scores above the clinical cut-off point and was therefore named the *clinical restraint* cluster. Cluster B4 (*n* = 27; 5 %) had scores above the clinical cut-off point on all subscales; as such, the cluster was named the *clinical DE* cluster. Cluster B5 (*n* = 8; 1 %), which comprised only 8 individuals, had scores above the clinical cut-off points on all subscales, but with especially high scores on the shape and weight concern subscales. It was subsequently named the *clinical DE and high shape and weight concern* cluster. Finally, Cluster B6 (*n* = 20; 4 %) was defined as the *clinical DE and high restraint* cluster because it showed scores above the clinical cut-off on all subscales but with particularly high scores on restraint.

The *non-problematic*, *clinical shape and elevated weight concern*, and *clinical restraint* clusters for boys all had homogeneity coefficients below the desired value of 1.00. In contrast, the *clinical DE* cluster had a value above 1.00 and the clusters *clinical DE with high shape and weight concern* and *clinical DE with high restraint* both had coefficients exceeding 2.00.

### Gender comparison of cluster solutions

**Fig. 1:**
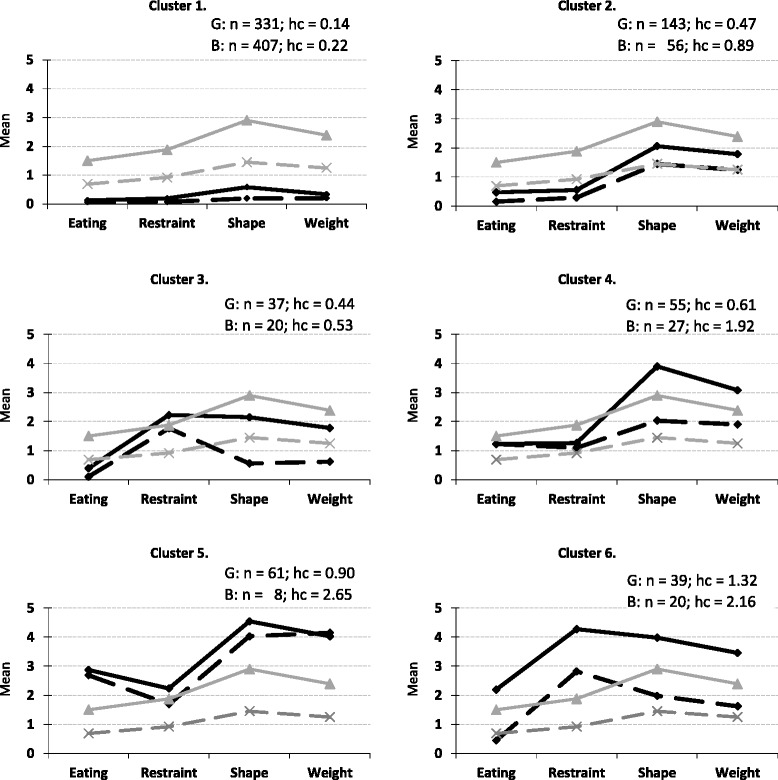
Graphical illustration (solid black lines for girls and dotted black lines for boys) of the typical DE-profiles (cluster means) in a 6-cluster solution. Solid grey lines and dotted grey lines indicate clinical cut-off scores for girls and boys, respectively. *Note.* hc = homogeneity coefficient

In Fig. 1, the mean profiles are presented graphically for the cluster solutions. Based on the ASED, examined by the CENTROID procedure, girls’ and boys’ clusters with the shortest ASED were paired. As shown in the figure, the girls’ scores tended to be higher than the boys’, but the patterns of DE indicators were rather similar between the genders.

Table 3 shows a comparison of the cluster sizes between the genders. As expected, significantly more girls than boys-with the exceptions of the *clinical restraint* and the *clinical DE with high restraint* clusters-were found in the problematic clusters, while significantly more boys than girls were found in the non-problematic cluster.

**Table 3:** Comparison of Cluster Sizes between the Genders

Clusters	Girls *n* (%)	Boys *n* (%)	Total *n* (%)
G1/B1	331 (50 %)	407 (76 %)	738 (61 %)
G2/B2	143 (21 %)	56 (10 %)	199 (16 %)
G3/B3	37 (6 %)	20 (4 %)	57 (5 %)
G4/B4	55 (8 %)	27 (5 %)	82 (7 %)
G5/B5	61 (9 %)	8 (1 %)	69 (6 %)
G6/B6	39 (6 %)	20 (4 %)	59 (5 %)
Total	666 (100 %)	538 (100 %)	1204 (100 %)

*Note:* Ns and (%) are significantly different at the *p* ≤ .05 level between girls and boys except for clusters G3/B3 and G6/B6, which did not differ significantly

### Cluster associations to the SCOFF categories

The trustworthiness of the cluster solutions was initially studied by examining whether cluster membership was associated with the categories of DE behaviors as measured by the SCOFF (≥2 or ≤ 1 “yes”). To examine this, we used the EXACON procedure in SLEIPNER, which enabled us to examine whether an observed pattern occurs significantly more often than would be expected by chance. These patterns are called *types*. In this procedure, we cross-tabulated the DE profiles and the two SCOFF categories (≥2 or and ≤1 “yes” responses on the SCOFF) and then performed exact tests on single cells in two-way contingency tables using hypergeometric probabilities.

#### Girls

The results revealed that girls in Clusters G4, G5 and G6 reported ≥2 “yes” responses on the SCOFF about two to four times as often as would be expected by chance (Cluster G4: observed = 15, expected = 8.2; *χ* ^2^ = 5.6, *p* < .02; Cluster G5: observed = 32, expected = 8.5; *χ* ^2^ = 64.5, *p* < .001; Cluster 6: observed = 23, expected = 5.5; *χ* ^2^ = 56.1, *p* < .001). About 29 % (n = 15/51), 60 % (n = 32/53) and 67 % (n = 23/34) of girls in Clusters G4, G5, and G6, respectively, had reported ≥2 “yes” responses on the SCOFF questionnaire.

#### Boys

Boys in Clusters B4 and B5 reported ≥2 “yes” responses about five times as often as would be expected by chance (Cluster B4: observed = 5, expected = 0.9; *χ* ^2^ = 5.6, *p* < .05; Cluster 5: observed = 5, expected = 0.3; *χ* ^2^ = 64.5, *p* < .001). About 20 % (n = 5/25 boys) and 71 % (n = 5/7) of the boys in Clusters B4 and B5, respectively, were found to belong report ≥2 “yes” responses on the SCOFF questionnaire.

### Cluster associations with emotion dysregulation and psychological health

We further confirmed the trustworthiness of the cluster solutions by examining whether cluster membership was associated with emotion dysregulation, depression, and self-esteem. Tables 4 (for girls) and 5 (for boys) present the results of one-way analyses of variance conducted to evaluate the differences among the clusters.

**Table 4:** N of 2& > yes and less than 2 yes to the SCOFF and M (SD) for emotion dysregulation (DERS), depression and self-esteem for girls’ cluster profiles

	G1 Non-problematic	G2 Elevated shape & weight concern	G3 Clinical restraint	G4 Clinical shape & weight concern	G5 Clinical DE, high shape & weight concern	G6 Clinical DE, high restraint	Total	*F* *	*η* ^2^	Post-hoc**
SCOFF N of 2& > “yes”/less than 2”yes”	9/309	14/117	7/27	15/36	32/21	23/11	100/521	–	–	–
DERS Global	70.75 (18.41)	84.39 (21.02)	72.46 (13.39)	92.91 (23.30)	101.57 (23.74)	103.71 (23.13)	80.50 (23.17)	(5, 92.48) =31.02	.26	G1<G2, G4, G5,G6 G2<G5,G6 G3<G4,G5,G6
Awareness	15.12 (4.70)	17.18 (4.22)	15.47 (4.40)	16.42 (4.64)	17.23 (4.58)	18.00 (3.67)	16.08 (4.61)	(5, 567) =6.10	.26	G1< G2, G5,G6
Clarity	9.81 (3.71)	12.44 (4.21)	10.83 (4.00)	13.02 (4.43)	14.45 (3.73)	14.19 (4.22)	11.38 (4.30)	(5, 598) =23.70	.17	G1< G2, G4, G5,G6 G2<G5; G3<G4,G6
Goals	11.57 (4.75)	13.05 (4.73)	11.18 (3.66)	15.06 (4.83)	16.43 (4.56)	15.80 (4.94)	12.87 (5.0)	(5, 371.54) =24.48	.13	G1<G2, G4, G5,G6 G2<G5,G6 G3<G4,G5,G6
Impulse	10.24 (4.11)	11.44 (4.87)	9.50 (3.21)	13.42 (5.10)	15.15 (5.73)	15.59 (5.97)	11.51 (4.97)	(5, 123.86) =19.13	.14	G1<G4, G5,G6 G2<G5,G6 G3<G4,G5,G6
Non-accept	10.35 (4.09)	13.08 (5.25)	10.97 (4.15)	15.13 (6.15)	16.37 (5.91)	15.18 (5.23)	12.22 (5.27)	(5, 116.24) =24.48	.18	G1<G2,G4, G5,G6 G2<G5; G3< G4,G5,G6
Strategies	13.64 (5.13)	17.08 (6.59)	13.27 (3.98)	18.94 (6.30)	21.16 (6.35)	21.38 (8.07)	15.93 (6.46)	(5, 111.60) =26.21	.20	G1<G2, G4, G5, G6 G2<G5, G6; G2>G3 G3<G4,G5,G6
CEDS-C Depression	13.79 (9.18)	21.71 (11.26)	14.03 (7.22)	24.35 (9.82)	30.17 (10.99)	29.41 (12.45)	18.80 (11.63)	(5, 118.17) =39.29	.27	G1<G2,G4, G5,G6 G2>G3; G2<G4, G5, G6 G3<G4,G5,G6
Rosenberg Self-Esteem	4.05 (.67)	3.57 (.76)	3.88 (.69)	3.11 (.80)	2.91 (.71)	3.09 (.81)	3.70 (.83)	(5, 645) =44.37	.26	G1>G2, G4,G5,G6 G2>G5, G4, G6 G3>G4,G5,G6

*Note*: *All *F*-values are significance at *p* < .001; **All post-hoc tests are significant at *p* < .05

**Table 5:** N of 2& > yes and less than 2 yes to the SCOFF and M (SD) for emotion dysregulation (DERS), depression and self-esteem for boys” cluster profiles

	B1 Non-problematic	B2 Clinical shape & elevated weight concern	B3 Clinical restraint	B4 Clinical DE	B5 Clinical DE, high shape & weight concern	B6 Clinical DE, high restraint	Total	F*	*η* ^2^	Post-hoc**
SCOFF N of 2& > “yes”/less than 2”yes”	4/389	3/50	2/16	5/20	5/2	1/19	20/496	–	–	–
DERS Global	63.46 (18.33)	79.0 (25.24)	57.33 (11.59)	88.88 (19.97)	111.33 (19.86)	73.17 (17.67)	66.73 (20.60)	(5, 344) =13.47	.16	B1<B2, B4, B5 B2>B3;B3<B4, B5 B5>B6
Awareness	15.25 (24.97)	16.46 (4.75)	12.76 (4.29)	16.71 (4.01)	21.17 (5.95)	16.47 (5.32)	15.48 (4.98)	(5, 433) =3.57	.04	B1<B5; B3<B5
Clarity	8.61 (3.61)	10.88 (4.23)	7.65 (2.57)	12.04 (3.14)	13.67 (3.56)	10.76 (3.93)	9.13 (3.81)	(5, 469) =10.0	.10	B1<B2, B4, B5 B2>B3; B3<B4, B5
Goals	9.87 (4.17)	12.36 (4.78)	9.47 (4.05)	14.55 (4.54)	16.50 (4.36)	11.27 (3.83)	10.45 (4.43)	(5, 436) =9.13	.09	B1<B2, B4, B5 B3<B4, B5
Impulse	9.36 (4.08)	12.43 (5.64)	7.76 (1.75)	13.36 (4.94)	15.50 (4.32)	10.31 (3.20)	9.93 (4.44)	(5, 32.17) =10.25^a^	.12	B1<B2, B4, B5 B2>B3; B3<B4, B5
Non-accept	9.26 (3.92)	12.13 (4.83)	9.38 (3.18)	13.05 (4.89)	15.00 (4.98)	10.94 (4.20)	9.89 (4.26)	(5, 431) =9.11	.10	B1<B2, B4, B5 B3<B5
Strategies	12.69 (4.97)	16.14 (7.01)	11.18 (3.11)	19.33 (5.71)	21.71 (4.72)	13.88 (3.69)	13.49 (5.51)	(5, 34.48) =12.21^a^	.14	B1<B2, B4, B5 B2>B3; B3< B4, B5 B4>B6; B5>B6
CEDS-C Depression	9.22 (8.09)	17.19 (12.31)	7.07 (6.04)	18.57 (9.55)	27.67 (11.04)	12.73 (7.21)	10.77 (9.33)	(5, 29.69) =15.53	.16	B1<B2, B4, B5; B2>B3 B3< B4, B5; B5>B6
Rosenberg Self-Esteem	4.35 (.65)	3.72 (.84)	4.52 (.45)	3.69 (.76)	2.80 (.86)	4.01 (.69)	4.22 (.74)	(5, 503) =19.69	.16	B1>B2, B4, B5 B2< B3; B2>B5 B3>B4, B5; B4>B5 B5<B6

*Note*: ^a^ Emotional impulse control and emotional strategies are reported with Welch’s test. *All *F*-values are significant at *p* < .001, except Awareness (*p* < .01). **All post-hoc tests are significant at *p* < .05

#### Girls

Tukey’s post-hoc tests revealed that, as expected, girls in the *non-problematic* cluster (Cluster G1) had significantly lower scores than did girls in the other clusters (with the exception of the *clinical restraint* cluster) on the DERS total score. This indicated that these girls had greater emotion regulation competency. They also showed significantly lower scores for depression and higher self-esteem. The *elevated shape and weight concern* cluster (Cluster G2) consistently scored higher than did the *non-problematic* cluster (Cluster G1) on all variables, except for emotional impulse control, where there was no significant difference between the two clusters.

Interestingly, the *clinical restraint* cluster (Cluster G3) did not score significantly differently from the *non-problematic* cluster on any variable (with the exception of the mean SCOFF scores).

The *clinical shape and weight concern* cluster (Cluster G4) significantly differed from the *non-problematic* cluster on all variables, except for emotional awareness. Notably, the *elevated-* and *clinical shape and weight concern* clusters (i.e., Clusters G2 and G4, respectively) only differed in terms of their mean scores on the SCOFF, which corresponds with the differing degree of severity expected between these two clusters.

With respect to emotional impulse control, the two *clinical DE* clusters (Cluster G5 and G6), which had the most severe DE, had significantly higher scores than did all of the other clusters; this indicated that they had more problems with emotion dysregulation. Further, both *clinical DE* clusters had significantly higher mean scores on the SCOFF questionnaire than did the remaining clusters. The *clinical DE with high restraint* cluster (Cluster G6) also showed significantly higher depression scores and significantly lower self-esteem scores than did the others, except the *clinical shape and weight concern* and *clinical DE with high shape and weight concern* clusters, indicating high levels of depressive thoughts and low self-esteem among the clusters with most problematic DE.

#### Boys

Tukey’s post-hoc tests showed that, as expected, the boys in the *non-problematic* cluster (Cluster B1) had significantly lower global DERS scores and most DERS subscale scores than did all of the other clusters (with the exception of the *clinical restraint* [Cluster B3] and *clinical DE with high restraint* clusters [Cluster B6]). Furthermore, with respect to the mean scores on the SCOFF, the *non-problematic* cluster showed significantly lower scores than did all of the other clusters except for the *clinical restraint* cluster, thus indicating fewer problems with DE. The boys’ *clinical restraint* cluster did not differ in any way from the *non-problematic* cluster. Furthermore, the *non-problematic* and *clinical restraint* clusters reported significantly lower scores for depression and significantly higher scores for self-esteem than did the other clusters, except for the *clinical DE with high restraint* cluster.

The *clinical shape with elevated weight concern* cluster (Cluster B2) showed significantly higher scores on all variables than did the *non-problematic* cluster, with the exception of emotional awareness. The *clinical DE* cluster (Cluster B4) differed from the *non-problematic* cluster on all variables, but when compared to the *clinical DE with high shape and weight concern* cluster (Cluster B5), it exhibited a lower SCOFF score and higher scores on self-esteem. The *clinical DE with high restraint* cluster (Cluster B6) did not differ significantly from the *non-problematic* cluster on any variable, with one exception: namely, it had a higher score on the SCOFF, indicating more severe DE. Cluster B6’s scores also significantly differed from those on the *clinical DE with high shape and weight concern* cluster, showing fewer problems with emotion dysregulation and depressive thoughts, better emotional strategies, and higher self-esteem.

## Discussion

Our results revealed six typical DE patterns that differed between girls and boys. The clusters contained reasonably homogeneous groups (especially among girls) and the differences between the clusters in the various validation variables were (in many cases) large and generally followed the expected directions. Although some of the DE patterns, such as the *non-problematic* (Cluster 1), the *elevated shape and weight/clinical shape and weight concern* (Cluster 2) and *clinical DE with high shape and weight concern* (Cluster 5) were similar between girls and boys, the remaining three patterns clearly differed. As expected, both boys and girls with the *non-problematic* DE patterns reported the highest levels of psychological health, including low levels of emotion dysregulation and depression and high self-esteem, when compared to their counterparts with more problematic DE patterns. In contrast, the adolescents with DE patterns above the clinical cut-offs tended to report higher levels of emotion dysregulation, more depressive symptoms, and lower self-esteem than did those with DE patterns below the clinical cut-offs; this aligned with our expectations.

Most of the girls (50 %) and boys (76 %) included in our sample had non-problematic DE patterns. Only around 3 % of girls and 1 % of boys with the non-problematic DE pattern reported ≥2 “yes” responses on the SCOFF. Although significantly more boys than girls had the non-problematic pattern, this was expected and in line with previous research (e.g., [19]).

While it is gratifying that a majority of studied adolescents did not report DE, rather large proportions of girls (29 %) and boys (24 %) still fell into clusters with DE patterns with at least one indicator above the clinical cut-off suggested by Ekeroth and Birgegard [11]. These prevalence rates are within the range found in previous research. Specifically, reported prevalence rates for girls vary from 30 % among Israeli girls (*M*_age_ = 14.7; [23]) to 52 % among Finnish girls (*M*_age_ = 14.9; [18]) as well as 56 % of girls in a large sample (over 40,000) of 9th and 12th grade American female adolescents (Croll et al., 2002). The prevalence rates of DE for boys have been found to vary as well, at 15 % as reported by Herpertz-Dahlmann et al. [20] in a German sample of 1,895 11- to 17-year-old adolescents; 17 % as reported by Hautala et al. [18] in a Finnish sample of adolescent boys aged 15; 25 % as reported by Lavender et al. [26] in a study of male undergraduates; and 29 % as reported by Croll et al. (2002) among American adolescents. The main reason for the varying DE prevalence rates across these studies is probably that DE can be defined and measured in numerous different ways. In some studies, DE is defined via specific DE behaviors as measured by the SCOFF questionnaire (e.g., [19, 20]) or using items specifically designed for that study (Croll et al., 2002), while in the present study, we used both the SCOFF questionnaire and the EDE-Q to provide a broader picture of DE.

Surprisingly, adolescents with the *clinical restraint* DE pattern generally did not differ from the *non-problematic* pattern in terms of emotion dysregulation, depression, or self-esteem and reported better mental health compared to the other problematic clusters. These clusters tended to be small (37 girls, or 6 % of all girls, and 20 boys, or 4 % of all boys) and homogeneous, indicating that the individuals included in the clusters scored alike on all four DE indicators. These results seem to point to the fact that clinical-level restraint does not, on its own, have a significant impact on adolescents’ psychological health. One possible explanation for these results is that the boys and girls in this cluster are comparable to “non-disordered obese” or overweight “jolly fat” individuals ([22], p. 635), who, despite their severe overweight, do not have the accompanying depressive thoughts common among obese and overweight individuals. It is possible as well that the high self-esteem reported by both genders with *the clinical restraint* DE pattern plays an important role, since high self-esteem has been found to be an important protective factor for the negative effects of DE for both girls and boys in a number of studies (e.g., Croll, Neumark-Sztainer, Story & Ireland, 2002; Micali et al., 2015). Another possible explanation is that adolescents with this pattern are more aware of what they eat than adolescents in general [33]. It would be informative to follow-up on adolescents with the *clinical restraint* DE pattern to determine whether they would continue to be emotionally well-regulated and show good mental health despite their continually elevated weight and shape concerns and restraint levels, or whether the restraint is merely temporary and either develops into healthy eating habits or worsens over time.

The most problematic DE patterns for girls were the *clinical DE with high shape and weight concern* pattern (Cluster G5) and the *clinical DE with high restraint* pattern (Cluster G6); specifically, they showed the worst emotion dysregulation, depression, and self-esteem. Furthermore, the two clusters had the highest proportions of girls with ≥2 “yes” responses on the SCOFF (60 % and 67 % in Cluster G5 and G6, respectively).

The *clinical DE with high restraint* cluster showed worse emotion dysregulation than did the *elevated shape and weight concern* cluster (G2) on all variables except for emotional awareness, emotional clarity, and non-acceptance of emotions. Although being aware of one’s (negative) emotions might cause elevated suffering, girls with clearly problematic DE might be unable to optimize their emotions to ensure appropriate responses [1]; however, but this does not suggest that they are not able to optimize their (negative) emotions at all. Why the non-acceptance of one’s emotions did not differ between the *clinical DEB with high restraint* cluster and the *elevated shape and weight concern* cluster is unclear, and should be studied further. Although the girls in Clusters G5 and G6 scored rather similar on emotion dysregulation, self-esteem, and depression, their significant differences with regard to restraint might indicate different developmental trajectories of DE that may need different intervention strategies. It should be noted that the lowest self-esteem of all clusters was found among girls with the *clinical DE with high shape and weight concern* pattern (Cluster G5), which is in line with findings that exaggerated views of shape and weight are strongly related with low self-esteem [10].

Among boys, one clearly problematic DE pattern emerged-the *clinical DE with high shape and weight concern* pattern (Cluster B5). Although this DE pattern was almost identical to the girls’ pattern (Cluster G5), significantly fewer boys had this pattern (*n* = 8 [1 %] compared to 61 [9 %] of girls). Furthermore, this DE pattern was the only one (compared to the two highly problematic DE patterns for girls) that clearly showed clinical signs of DE among boys, as five of the boys in the cluster (71 %) reported ≥2 “yes” responses on the SCOFF questionnaire. The DE patterns significantly differed from all other clusters among the boys, having higher scores on emotion dysregulation and depression and the lowest self-esteem scores of all clusters (i.e., both girls and boys). This last point is noteworthy considering that girls otherwise tended to have a lower self-esteem score than did boys in this study. Previous research has suggested that men who experience emotion dysregulation may use DE to modulate or escape unwanted emotions [25], which, in combination with low self-esteem, may be devastating to their mental health.

Surprisingly, boys with the *clinical DE with high restraint* pattern (Cluster B6), despite having scores above the cut-offs on three of the four DE indicators, reported rather efficient emotional regulation strategies, low depression, and relatively good self-esteem. Furthermore, only one in 20 boys with this problematic DE pattern reported ≥2 “yes” responses on the SCOFF questionnaire, further indicating that these boys seemed to have a lower risk for DE. However, it would be important to follow up on the boys with this DE pattern in order to confirm whether high self-esteem in combination with good emotion regulation plays a decisive role in how the pattern develops. In other words, it may be that the high scores on these three DE indicators are temporary in this cluster, and do not develop into clinical levels of DE.

Although Clusters G4 and B4 (*clinical shape and weight concern* among girls and *clinical DE* among boys, respectively) had scores above the clinical cut-offs on two of the four DE indicators, these clusters did not differ cardinally from the less problematic clusters. Nevertheless, these patterns showed some important signs (e.g., significantly more boys [20 % within the cluster] and girls [29 % within the cluster] reporting ≥2 “yes” responses on the SCOFF than could be expected by chance, elevated scores on emotion dysregulation and depression, rather lower self-esteem) that suggest that this pattern may become more problematic in the future. In other words, this pattern may be considered a “pre-clinical” or “turning point” pattern requiring special attention from both healthcare personnel and parents. However, more results are needed to confirm this.

In general, the boy clusters appeared to be less distinct than the girl clusters. This could, certainly, be because of the difficulties in measuring overall DE (e.g., [7]) or to the specific difficulties in measuring DE and emotion dysregulation in boys [33]. Additionally, it might be simply that boys’ clusters were smaller, thus diminishing the statistical power. Another alternative is that the clinical cut-offs suggested by Ekeroth and Birgegard [11] are too low for boys, since, as discussed above, the DE pattern that had indicators above the clinical cut-offs (Cluster B6) was not confirmed as problematic according to the validation variables. Although boys tend to have less severe DE symptoms, and therefore have correspondingly lower clinical cut-offs, more research may be needed to fine-tune the current cut-offs for them. On the other hand, when using the EDE-Q as a screening tool, it would be valuable to use cut-offs with high sensitivities to avoid passing over adolescents who might be at risk of developing DE or are already experiencing it.

### Strengths and limitations

This study has several strengths. First, the sample included in the present study was a large, representative, gender-diverse sample of community-dwelling adolescents. Second, this is, to our knowledge, the first study to examine typical patterns of DE among adolescents of both genders. Third, we used an advanced form of cluster analysis that allowed us to validate our cluster solutions in a more sophisticated way, including calculation of the variance explained and homogeneity coefficients.

Nevertheless, this study also has some limitations. First, the data analyzed in the study were only from self-reports. The main shortcomings of such data concern shared-method variance, conscious distortion, social comparison, and situational and contextual factors that to some degree limit our drawing stronger conclusions. To verify the results, a multi-method approach that involves diagnostic interviews, parent reports, and hospital records would be advantageous in future studies. Second, although we used a large sample, all of the participants were drawn from one municipality in Sweden, meaning that potential local bias may exist; however, it should be noted that this municipality was, in many respects, representative of Sweden as a whole.

## Conclusions

Despite the limitations, this study had a number of important findings. First, we found six typical DE patterns among both girls and boys. These patterns suggest an alternative way of representing the relationship structure among the various DE indicators. Four of the six girls’ clusters and five of the boys’ clusters had scores on at least one DE indicator above recommended clinical cut-offs. However, the most problematic clusters comprised adolescents who reported scores above the clinical cut-offs on all DE indicators in combination with severe psychological problems, including emotion dysregulation, high levels of depression, low self-esteem, and higher proportions of adolescents with ≥ 2 “yes” responses on the SCOFF questionnaire. Unexpectedly, both girls and boys who reported restraint subscale scores above the cut-offs had psychological health comparable to the non-problematic DE pattern. Because several different problematic DE patterns emerged, it is likely that they would require different paths back to a healthy diet. Longitudinal studies that follow-up on the emerged DE patterns in order to study their developmental trajectories in combination with important psychological variables (e.g., self-esteem) would be of great importance in the future. By expanding our knowledge of the patterns, the detection and prevention of DE can be improved, which in turn would reduce the likelihood of DE problems becoming chronic into adulthood [39].

## Abbreviations

ASED: Average squared Euclidean Distances
DE: Disordered Eating
DERS: Difficulties in Emotions Regulations Questionnaire
EDE-Q: Eating Disorders Examination Questionnaire
EESS: Explained Error Sum of Squares
